# Differential effects of acute cerebellectomy on cough in spontaneously breathing cats

**DOI:** 10.1371/journal.pone.0253060

**Published:** 2021-06-21

**Authors:** M. Nicholas Musselwhite, Tabitha Y. Shen, Melanie J. Rose, Kimberly E. Iceman, Ivan Poliacek, Teresa Pitts, Donald C. Bolser

**Affiliations:** 1 Department of Physiological Sciences, College of Veterinary Medicine, University of Florida, Gainesville, Florida, United States of America; 2 Department of Neurological Surgery and Kentucky Spinal Cord Injury Research Center, College of Medicine, University of Louisville, Louisville, Kentucky, United States of America; 3 Comenius University in Bratislava, Jessenius Faculty of Medicine in Martin, Institute of Medical Biophysics, Martin, Slovak Republic; National Yang-Ming University, TAIWAN

## Abstract

The role of the cerebellum in controlling the cough motor pattern is not well understood. We hypothesized that cerebellectomy would disinhibit motor drive to respiratory muscles during cough. Cough was induced by mechanical stimulation of the tracheobronchial airways in anesthetized, spontaneously breathing adult cats (8 male, 1 female), and electromyograms (EMGs) were recorded from upper airway, chest wall, and abdominal respiratory muscles. Cough trials were performed before and at two time points after total cerebellectomy (10 minutes and >1 hour). Unlike a prior report in paralyzed, decerebrated, and artificially ventilated animals, we observed that cerebellectomy had no effect on cough frequency. After cerebellectomy, thoracic inspiratory muscle EMG magnitudes increased during cough (diaphragm EMG increased by 14% at 10 minutes, *p* = 0.04; parasternal by 34% at 10 minutes and by 32% at >1 hour, *p* = 0.001 and 0.03 respectively). During cough at 10 minutes after cerebellectomy, inspiratory esophageal pressure was increased by 44% (*p* = 0.004), thyroarytenoid (laryngeal adductor) muscle EMG amplitude increased 13% (*p* = 0.04), and no change was observed in the posterior cricoarytenoid (laryngeal abductor) EMG. Cough phase durations did not change. Blood pressure and heart rate were reduced after cerebellectomy, and respiratory rate also decreased due to an increase in duration of the expiratory phase of breathing. Changes in cough-related EMG magnitudes of respiratory muscles suggest that the cerebellum exerts inhibitory control of cough motor drive, but not cough number or phase timing in response to mechanical stimuli in this model early after cerebellectomy. However, results varied widely at >1 hour after cerebellectomy, with some animals exhibiting enhancement or suppression of one or more components of the cough motor behavior. These results suggest that, while the cerebellum and behavior-related sensory feedback regulate cough, it may be difficult to predict the nature of the modulation based on total cerebellectomy.

## Introduction

Cough is vital to maintain a clear airway and a functional respiratory system. It is elicited through mechanical and a variety of chemical stimuli applied to the airways including capsaicin and other TRPV1 agonists, acids, bradykinin, and histamine [1,2]. A*δ* fibers transmit information related to punctate mechanical stimuli and acids, via the recurrent laryngeal and superior laryngeal branches of the vagus nerve, and terminate in the nucleus of the solitary tract (NTS), where they synapse on second-order interneurons [3,4]. These second order neurons then synapse with various respiratory areas in the brainstem, including the ventral respiratory group, retrotrapezoid nucleus/parafacial respiratory group, midline raphé, nucleus ambiguus, and pontine respiratory group [5–7]. While cough is elicited by vagal pathways, preliminary evidence suggests that other afferent feedback and/or input from areas of the brain responsible for movement coordination could play a key role [8,9].

Current theories of the cerebellum suggest that motor learning is required to create appropriate internal models under varying proprioceptive and exteroceptive states [10]. In locomotion, it is classically understood that sensory feedback arising from muscles spindles and Golgi tendon organs via spinocerebellar pathways [11] is vital to generating these internal models [12–14]. For upper airway behaviors, afferent information from the larynx and tracheobronchial tree is represented in the cerebellum [15,16], and perturbation of cerebellar regions can modify breathing, presumably through synaptic connections with the respiratory control network (RCN) [17–19]. For example, Glasser et al. [17] demonstrated that removal of the cerebellum inhibited breathing. More recently, Xu and Frasier [18] demonstrated that electrical stimulation of the fastigial nucleus modulated respiratory phase timing, and that cerebellar ablation attenuated the hypoxic ventilatory response.

The role of the cerebellum in airway protection is not wholly understood. Only one previous study has investigated the effects of cerebellectomy on cough in decerebrate cats [20]. Removal of the cerebellum reduced cough number and expiratory drive with no significant changes in inspiratory activity. However, a paralytic agent was used that would have resulted in little or no phasic somatic afferent feedback during coughing, likely confounding the effect of cerebellectomy. There is also a case report [21] of cerebellar ataxia increasing cough excitability, leading to cough-related syncope.

To further investigate the influence of cerebellectomy on cough, we examined the effect of cerebellectomy on cough motor drive in anesthetized and unparalyzed animals. We hypothesized that, when retaining sensory feedback, cough motor drive would increase after cerebellectomy. This would contrast the results of Xu and colleagues [20], likely resulting from disinhibition of central cough pathways.

## Methods

Experiments were performed on 9 spontaneously breathing adult cats (8 male, 1 female), with an average weight of 5.2 kg (standard deviation = 0.7 kg). The protocol was approved by the University of Florida Institutional Animal Care and Use Committee (IACUC). Anesthesia was initially induced with sevoflurane (4.5%), then the animals were weaned onto sodium pentobarbital (25 mg/kg, i.v.). Based on forelimb withdrawal reflex and jaw tone, supplemental doses were given as needed in 0.1–0.3 mL i.v. boluses. A single dose of atropine sulfate (0.054 mg/kg, i.v.) was given at the beginning of surgery to minimize airway secretions. The femoral vein and artery were cannulated to deliver drugs and record blood pressure, respectively. Arterial blood pressure and end-tidal CO_2_ were continuously recorded, and arterial blood gas samples were obtained every hour. Body temperature was maintained at 37.5 ± 0.5°C with a homeothermic pad and rectal temperature probe.

Animals were tracheostomized and a bifurcated tracheostomy tube was placed to permit simultaneous end tidal gas recording and mechanical stimulation of the tracheobronchial airway to elicit coughing. Bipolar fine wire electromyograms (EMGs) were placed intramuscularly to record respiratory related behaviors using the method of Basmajian and Stecko [22]. EMGs from the sternocostal diaphragm (dorsal to the xiphoid process), bilateral parasternal, bilateral internal abdominal oblique, thyroarytenoid, and posterior cricoarytenoid (PCA) muscles were evaluated during cough and breathing. EMG signals were amplified using Grass P511 amplifiers and band-pass filtered (300–10,000 Hz) before being digitized with Spike 2 version 9 (Cambridge Electronic Design; United Kingdom). EMG channels were rectified and smoothed with a 50 ms time constant prior to analysis. A lead II electrocardiogram (ECG) was also placed on the animal [23], similarly amplified and digitized, with a band pass filter set to 3-3000Hz. Esophageal pressure was measured via a small balloon inserted through the mouth into the midthoracic esophagus. EMG and ECG signals were sampled at 25,000 Hz and pressure signals at 500 Hz.

Cough was stimulated via a flexible plastic cannula inserted into the tracheal tube. Mechanical stimulation of the intrathoracic trachea was accomplished by inserting and rotating this cannula at approximately 2 Hz for periods of 10–15 seconds. In the control period prior to cerebellectomy, an adaptation protocol consisting of 20 sequential mechanical stimulation trials was conducted, separated by ~1 minute [24]. Following this protocol, the cough response remains stable for several hours in this preparation.

A 1% lidocaine solution was injected into the external auditory canals to locally block the auricular nerves prior to placing the animal in a stereotaxic head stage. All coughs were stimulated while the animal was in the stereotaxic frame. After control coughs were recorded, a craniotomy was performed, and the cerebellum was removed by aspiration. The surface of the brainstem was covered with mineral oil. Post cerebellectomy cough trials were performed >1 hour after cerebellectomy with a subset of 5 animals also subject to 10 minute post-cerebellectomy trials. Tracheobronchial cough elicited after a period of rest often results in the initial trial being unrepresentative of subsequent trials, typically resulting in a more subdued response. For this reason, three trials were performed at each timepoint with a 60 second intertrial interval, and the first trial was omitted for analysis purposes.

**Fig 1:**
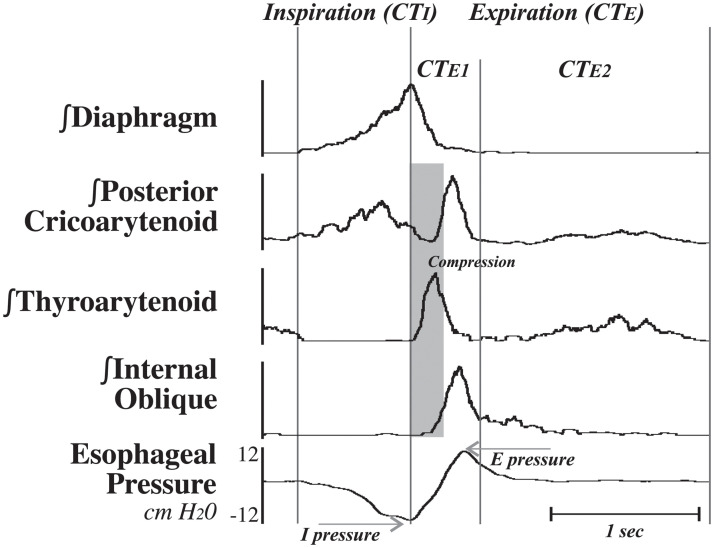
Representative example with integrated EMG traces used to define the phases of a single cough. The inspiratory phase of cough (CT_I_) was defined as the period of time between the start of diaphragm activity to the peak of the integrated diaphragm EMG, while the expiratory period (CT_E_) consisted of the time between the peak of the diaphragm to the subsequent start of activity on the diaphragm. CT_E_ was subdivided into an active expiratory phase (CT_E1_) beginning at the end of CT_I_ and continuing to the end of the ballistic effort on the internal oblique EMG, and a passive expiratory phase (CT_E2_) beginning at the end of CT_E1_ and the start of the next CT_I_. The *theoretical* compression phase of cough is highlighted in grey (all animals had a tracheostomy), corresponding to the ballistic activity of the thyroarytenoid.

Cough was confirmed by observing sequential and large ballistic-like EMG bursting patterns in inspiratory muscles, thyroarytenoid, and the internal abdominal obliques [25–27] and rapidly rising inspiratory and expiratory esophageal pressure changes that were much larger than those observed during eupneic breathing (Fig 1). Animals were euthanized via an i.v. overdose of sodium pentobarbital and, upon cessation of respiratory activity, a 3 cc bolus of saturated potassium chloride. Euthanasia was confirmed with a 5 minute absence of cardiac activity.

The inspiratory (CT_I_) phase of cough was defined as the period from the onset to the peak of activity of the diaphragm EMG. The active expiration component (CT_E1_) began when the inspiratory phase ended and lasted until the ballistic activity on the internal abdominal oblique EMG subsided. The passive expiration (CT_E2_) lasted from the end of the CT_E1_ phase until the onset of the inspiratory phase of the following cough. Similar phase timings were recorded for the eupneic period of breathing that preceded the cough stimulus. This period consisted of the four breaths preceding a cough trial. The inspiratory (T_I_) and expiratory (T_E_) phases of breathing were measured using the presence (T_I_) or absence (T_E_) of activity on the diaphragm EMG. The total cycle time (T_TOT_) measured the duration of one breath by adding T_I_ to T_E_. During this period, cardiovascular parameters were also measured. Heart rate was measured using the interval between R peaks on the ECG [23]. Systolic, diastolic, pulse, and mean arterial pressures were measured per beat. The data collected during this period was aggregated for each condition in each animal.

Cough and breathing related EMG magnitudes were normalized as a percent of the maximum for each muscle in each animal. Coughs that occurred with additional airway protective behaviors (i.e., swallow) were excluded from this analysis. Additionally, any cough generating an esophageal pressure less than 10% of that epoch’s maximum was excluded. A mean ± standard deviation was calculated for each animal, and then averaged for each condition across animals. Conditions were compared using a Linear Mixed Effects Model. All hypothesis testing measures had α = 0.05. Statistical analysis was performed in R using RStudio IDE [28,29]. Data was processed using the tidyverse package [30]. Statistical models were fit using the nlme package [31] with a fixed effect of condition and random effect of animal. Heterogeneous variance between conditions was accounted for when necessary. Post hoc testing was performed using the emmeans package [32] with a containment method of calculating degrees of freedom and using a Tukey HSD to correct for multiple comparisons. Additionally, Pearson’s product moment correlations (*r*) were calculated comparing all amplitude and duration measures to determine relationships using SPSS statistical software.

## Results

Tracheal stimulation resulted in bouts of repetitive cough during control and following removal of the cerebellum (measured 10 minutes and >1 hour afterward), and a total of 280 coughs were analyzed. There was no significant difference of cough frequency between conditions [F(2,12) = 0.68; *p* = 0.5].

### Cough amplitude and duration

Table 1 reports data measuring during cough trials before and after cerebellectomy. There was a significant effect of condition on percent of maximum diaphragm [F(2,12) = 4.28; *p* = 0.04], parasternal [F(2,12) = 12.59; *p* = 0.001] and thyroarytenoid [F(2,12) = 6.16; *p* = 0.014] EMG amplitude during cough, and on peak inspiratory esophageal pressure [F(2,12) = 8.48; *p* = 0.005]. Post hoc analysis revealed significant amplitude changes at 10 minutes post-cerebellectomy compared to control: the diaphragm increased by 14% (*p* = 0.04), parasternal increased by 34% (*p* < 0.001), thyroarytenoid (i.e., laryngeal adductor) increased by 13% (*p* = 0.04) and esophageal pressure by 44% (*p* = 0.004). At >1 hour post-cerebellectomy compared to control, parasternal EMG amplitude was still increased by 32% (*p* = 0.03), and while the thyroarytenoid was 32% higher than control, that effect was not significant (*p* = 0.1). By the >1 hour time point there was also no longer a change in inspiratory esophageal pressure (*p* = 0.99).

**Table 1:** Raw means and standard deviations for cough. (A.) EMG amplitudes (% of maximum), esophageal pressures (cm H_2_0), (B.) phase durations (ms) and cough frequency (Hz) for each animal and across animals for each condition.

**A. Amplitude**	**Condition**	**Animal #**	**Diaphragn**	**Parasternal**	**Internal Oblique**	**PCA**	**Thyroarytenoid**	**I pressure (cm H**_**2**_**0)**	**E pressure (cm H**_**2**_**0)**
	Control	1	70 ± 10	56 ± 9	83 ± 17	90 ± 7	47 ± 16	-13 ± 2	55 ± 20
	Control	2	48 ± 25	59 ± 20	25 ± 14	41 ± 10	19 ± 13	-9 ± 3	19 ± 11
	Control	3	74 ± 15	76 ± 14	64 ± 20	82 ± 13	56 ± 12	-11 ± 2	25 ± 8
	Control	4	48 ± 8	41 ± 11	29 ± 9	45 ± 4	70 ± 21	-8 ± 1	11 ± 6
	Control	5	44 ± 6	56 ± 5	54 ± 12	55 ± 11	50 ± 14	-4 ± 1	19 ± 5
	Control	6	80 ± 11	81 ± 13	55 ± 18	89 ± 2	31 ± 10	-20 ± 4	33 ± 13
	Control	7	59 ± 14	30 ± 7	59 ± 6	75 ± 15	42 ± 14	-1 ± 1	35 ± 9
	Control	8	41 ± 10	29 ± 8	23 ± 13	43 ± 19	8 ± 0	-5 ± 2	7 ± 4
	Control	9	41 ± 17	53 ± 11	46 ± 23	47 ± 16	20 ± 5	-14 ± 6	33 ± 12
	>1-hr post	1	78 ± 16	71 ± 18	62 ± 23	70 ± 9	72 ± 18	-10 ± 2	38 ± 22
	>1-hr post	2	66 ± 18	79 ± 16	59 ± 28	73 ± 22	58 ± 30	-10 ± 2	49 ± 23
	>1-hr post	3	41 ± 11	56 ± 9	27 ± 8	64 ± 8	52 ± 25	-5 ± 1	9 ± 3
	>1-hr post	4	68 ± 18	79 ± 24	71 ± 19	79 ± 11	70 ± 14	-10 ± 1	21 ± 5
	>1-hr post	5	47 ± 9	55 ± 8	76 ± 17	89 ± 8	54 ± 14	-4 ± 1	17 ± 4
	>1-hr post	6	68 ± 8	74 ± 7	44 ± 8	94 ± 3	28 ± 15	-17 ± 3	20 ± 5
	>1-hr post	7	71 ± 11	75 ± 15	36 ± 9	71 ± 8	44 ± 10	-6 ± 1	11 ± 3
	>1-hr post	8	49 ± 30	63 ± 24	43 ± 38	24 ± 4	38 ± 36	-6 ± 3	7 ± 5
	>1-hr post	9	46 ± 20	76 ± 15	50 ± 20	54 ± 13	31 ± 19	-16 ± 6	12 ± 2
	10-min post	5	62 ± 16	76 ± 12	68 ± 9	61 ± 7	53 ± 13	-6 ± 2	18 ± 3
	10-min post	6	84 ± 8	89 ± 9	67 ± 17	92 ± 3	56 ± 22	-26 ± 4	40 ± 14
	10-min post	7	76 ± 14	81 ± 14	77 ± 13	80 ± 7	61 ± 20	-7 ± 1	25 ± 6
	10-min post	8	47 ± 13	49 ± 13	55 ± 26	42 ± 21	9 ± 1	-8 ± 2	13 ± 6
	10-min post	9	50 ± 16	61 ± 12	42 ± 22	54 ± 21	34 ± 13	-16 ± 5	18 ± 6
**Average**	Control		56 ± 15	53 ± 18	49 ± 20	63 ± 21	38 ± 20	-9 ± 6	26 ± 15
	>1-hr post		59 ± 13	70 ± 9 *	52 ± 16	69 ± 21	50 ± 16	-9 ± 5	20 ± 14
	10-min post		64 ± 16 *	71 ± 16 ***	62 ± 14	66 ± 20	43 ± 21 *	-13 ± 9 **	23 ± 10

**B. Duration**	**Condition**	**Animal #**	**CT**_**I**_**(ms)**	**CT**_**E**_ **(ms)**	**CT**_**E1**_ **(ms)**	**CT**_**E2**_ **(ms)**	**CT**_**TOT**_ **(ms)**	**Frequency (Hz)**
	Control	1	637 ± 74	2528 ± 450	263 ± 48	2265 ± 443	3165 ± 441	0.24
	Control	2	801 ± 150	2008 ± 490	438 ± 87	1570 ± 470	2809 ± 565	0.42
	Control	3	765 ± 179	1109 ± 328	380 ± 23	729 ± 320	1874 ± 374	0.56
	Control	4	962 ± 235	2111 ± 549	548 ± 104	1563 ± 557	3073 ± 600	0.35
	Control	5	709 ± 138	1055 ± 150	451 ± 75	604 ± 145	1764 ± 235	0.59
	Control	6	764 ± 116	1536 ± 353	711 ± 122	825 ± 299	2300 ± 379	0.46
	Control	7	1152 ± 219	1297 ± 432	530 ± 54	767 ± 462	2449 ± 345	0.35
	Control	8	826 ± 287	925 ± 116	430 ± 97	495 ± 69	1751 ± 269	0.57
	Control	9	614 ± 167	1065 ± 363	588 ± 62	478 ± 326	1679 ± 484	0.61
	>1-hr post	1	1107 ± 363	1597 ± 643	274 ± 33	1324 ± 627	2705 ± 875	0.41
	>1-hr post	2	977 ± 239	1471 ± 423	321 ± 38	1151 ± 415	2448 ± 530	0.44
	>1-hr post	3	520 ± 255	789 ± 154	385 ± 51	405 ± 136	1309 ± 309	0.80
	>1-hr post	4	814 ± 116	1049 ± 257	543 ± 84	506 ± 268	1863 ± 291	0.51
	>1-hr post	5	755 ± 113	850 ± 113	349 ± 44	501 ± 114	1606 ± 186	0.66
	>1-hr post	6	660 ± 154	1043 ± 48	403 ± 96	640 ± 128	1702 ± 167	0.60
	>1-hr post	7	988 ± 90	1631 ± 198	611 ± 41	1020 ± 217	2619 ± 225	0.40
	>1-hr post	8	1607 ± 434	1519 ± 326	567 ± 219	952 ± 206	3126 ± 590	0.24
	>1-hr post	9	622 ± 148	893 ± 160	584 ± 83	309 ± 137	1515 ± 201	0.67
	10-min post	5	862 ± 115	985 ± 183	355 ± 43	630 ± 185	1847 ± 196	0.56
	10-min post	6	655 ± 134	1169 ± 244	636 ± 97	533 ± 247	1823 ± 339	0.58
	10-min post	7	859 ± 101	1301 ± 338	529 ± 66	772 ± 293	2161 ± 377	0.46
	10-min post	8	1241 ± 353	1334 ± 390	486 ± 127	848 ± 314	2575 ± 503	0.37
	10-min post	9	878 ± 232	1376 ± 507	636 ± 53	740 ± 532	2253 ± 583	0.45
**Average**	Control		803 ± 167	1515 ± 570	482 ± 130	1033 ± 620	2318 ± 590	0.46 ± 0.13
	>1-hr post		894 ± 329	1205 ± 345	449 ± 128	756 ± 362	2099 ± 636	0.53 ± 0.17
	10-min post		899 ± 212	1233 ± 159	528 ± 117	704 ± 124	2132 ± 312	0.48 ± 0.09

Tukey’s HSD test used to compare timepoints.

* = p < 0.05,

** = p < 0.01,

*** p < 0.001.

n = 9.

There were no significant changes in the amplitudes of the internal obliques [F(2,12) = 1.24; *p* = 0.3], posterior cricoarytenoid (PCA; i.e., laryngeal abductor) [F(2,12) = 0.4; *p* = 0.7], or expiratory esophageal pressure during cough [F(2,12) = 0.77; *p* = 0.5]. There were also no significant changes in cough phase durations: CT_I_ [F(2,12) = 0.64; *p* = 0.6], CT_E_ [F(2,12) = 1.21; *p* = 0.3], CT_E1_ [F(2,12) = 0.4; *p* = 0.7], CT_E2_ [F(2,12) = 1.12; *p* = 0.4], or CT_TOT_ [F(2,12) = 0.39; *p* = 0.7] (Table 1).

**Fig 2:**
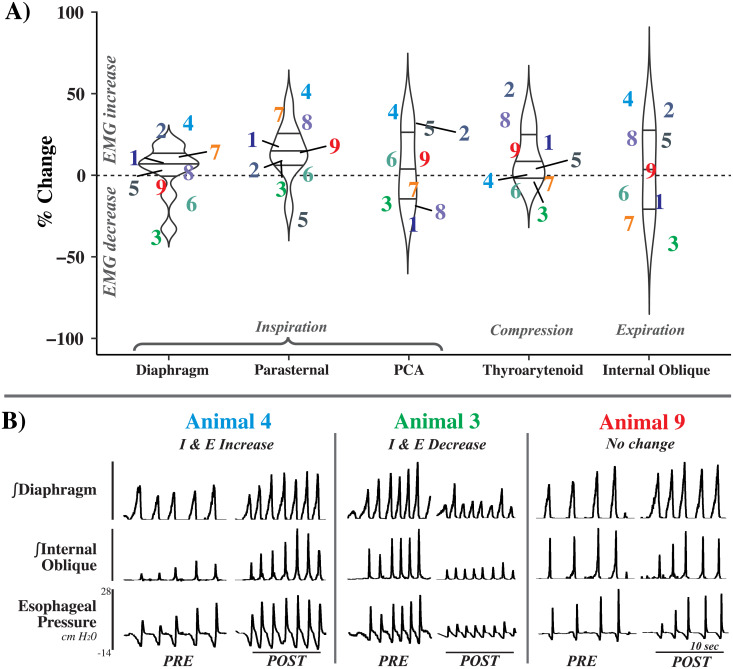
Animal-by-animal effects of cerebellectomy on cough. A) Percent change of maximum EMG amplitude >1 hour post-cerebellectomy during cough, plotted by animal number for the diaphragm, parasternal, posterior cricoarytenoid (PCA), thyroarytenoid, and internal oblique muscles. Note the spread for each muscle in the increasing and decreasing directions. B) Examples from 4 animals showing disparate responses to cerebellectomy. While there was a trend towards the enhancement of some cough related EMGs, particularly the inspiratory pump muscles (Animals 3 and 4), individual responses were heterogeneous, with some EMGs being suppressed (Animal 3) or not changing at all (Animal 9). n = 9.

Upon inspection of the physiological recordings and the measured data at the > 1 hour timepoint, it was apparent that the effect of cerebellectomy on cough varied greatly between animals; Fig 2 illustrates changes in cough features by animal.

### Breathing and cardiovascular measures

Table 2 reports data for breathing and cardiovascular measures. Significant condition effects were a decrease in respiratory rate [F(2,12) = 5.803; *p* = 0.02], an increase in T_E_ [F(2,12) = 4.959; *p* = 0.03], and an increase in T_TOT_ that did not reach significance [F(2,12) = 3.545; *p* = 0.06]. Post hoc analysis revealed significant changes 10 minutes post-cerebellectomy compared to control: a 24% decrease in respiratory rate (*p* = 0.02); the 62% increase in T_E_ and 32% increase in T_TOT_ did not reach significance (*p* = 0.06 and 0.06, respectively). At this 10-minute timepoint, there were no effects on end-tidal CO_2_ [F(2,12) = 1.538; *p* = 0.3], T_I_ [F(2,12) = 0.542; *p* = 0.6], or diaphragm EMG (percent maximum) activity during breathing [F(2,12) = 0.405; *p* = 0.7]. By the >1 hour timepoint, there were no significant effects on any breathing measures.

**Table 2:** Raw means and standard deviations of cardiorespiratory parameters measured preceding a cough trial. (A.) Respiratory parameters measured include respiratory rate (breaths/min), end-tidal CO_2_ (ETCO_2_, mmHg), T_I_, T_E_, and T_TOT_ phases of breathing (ms), and diaphragm EMG amplitude (% maximum). (B.) Cardiovascular measures were also recorded including systolic, diastolic, mean arterial pressure (MAP), and pulse pressure (PP) (mmHg) as well as heart rate (beats/min) preceding each cough trial.

**A. Eupnea**	**Condition**	**Animal #**	**Resp Rate (breath/min)**	**ETCO**_**2**_ **(mmHg)**	**T**_**I**_ **(ms)**	**T**_**E**_ **(ms)**	**T**_**TOT**_ **(ms)**	**Diaphram EMG (% max)**
	Control	1	21 ± 2	31 ± 1	1416 ± 214	1456 ± 170	2873 ± 294	46 ± 5
	Control	2	36 ± 1	22 ± 1	462 ± 49	1203 ± 53	1665 ± 88	51 ± 9
	Control	3	26 ± 1	35 ± 1	1167 ± 84	1180 ± 29	2346 ± 98	93 ± 5
	Control	4	21 ± 2	28 ± 1	1895 ± 481	978 ± 74	2873 ± 506	76 ± 4
	Control	5	26 ± 5	29 ± 2	956 ± 267	1416 ± 227	2372 ± 479	32 ± 6
	Control	6	26 ± 0	29 ± 1	762 ± 43	1531 ± 135	2293 ± 172	83 ± 8
	Control	7	14 ± 1	34 ± 0	1789 ± 109	2504 ± 351	4293 ± 423	91 ± 6
	Control	8	23 ± 0	31 ± 1	1601 ± 338	1005 ± 43	2607 ± 369	92 ± 2
	Control	9	31 ± 1	28 ± 0	540 ± 27	1423 ± 64	1964 ± 66	84 ± 5
	>1-hr post	1	12 ± 1	26 ± 0	1797 ± 85	3389 ± 267	5186 ± 333	96 ± 3
	>1-hr post	2	19 ± 1	34 ± 0	948 ± 68	2250 ± 222	3198 ± 242	91 ± 7
	>1-hr post	3	23 ± 0	29 ± 1	990 ± 180	1582 ± 198	2573 ± 55	82 ± 2
	>1-hr post	4	33 ± 2	24 ± 2	965 ± 133	879 ± 63	1845 ± 172	80 ± 8
	>1-hr post	5	43 ± 1	16 ± 0	773 ± 37	633 ± 26	1406 ± 53	29 ± 2
	>1-hr post	6	21 ± 1	27 ± 1	966 ± 97	1886 ± 147	2851 ± 215	88 ± 3
	>1-hr post	7	14 ± 2	33 ± 1	1149 ± 200	3310 ± 752	4458 ± 585	55 ± 9
	>1-hr post	8	20 ± 2	23 ± 0	1362 ± 156	1669 ± 157	3031 ± 293	87 ± 5
	>1-hr post	9	26 ± 0	26 ± 0	802 ± 87	1496 ± 53	2299 ± 109	97 ± 2
	10-min post	5	25 ± 2	23 ± 2	1204 ± 721	1201 ± 235	2405 ± 505	34 ± 27
	10-min post	6	16 ± 1	28 ± 1	907 ± 67	2752 ± 170	3659 ± 185	90 ± 4
	10-min post	7	14 ± 0	34 ± 0	976 ± 83	3232 ± 199	4208 ± 226	54 ± 3
	10-min post	8	14 ± 0	31 ± 1	1810 ± 59	2459 ± 168	4269 ± 182	96 ± 5
	10-min post	9	23 ± 1	26 ± 1	778 ± 45	1785 ± 90	2563 ± 115	91 ± 3
**Average**	Control		25 ± 6	30 ± 4	1177 ± 532	1411 ± 456	2587 ± 750	72 ± 23
	>1-hr post		23 ± 10	26 ± 5	1084 ± 320	1899 ± 955	2983 ± 1200	78 ± 22
	10-min post		19 ± 5*	29 ± 4	1135 ± 408	2286 ± 802	3421 ± 889	73 ± 27

**B. Cardio**	**Condition**	**Animal #**	**Systolic (mmHg)**	**Diastolic (mmHg)**	**MAP (mmHg)**	**PP (mmHg)**	**HR (beats/min)**
	Control	3	210 ± 6	149 ± 3	169 ± 4	61 ± 3	205 ± 2
	Control	4	145 ± 2	104 ± 1	118 ± 2	41 ± 1	202 ± 1
	Control	5	189 ± 5	127 ± 5	148 ± 5	61 ± 1	237 ± 5
	Control	6	159 ± 2	123 ± 2	135 ± 2	36 ± 1	210 ± 1
	Control	7	170 ± 4	124 ± 3	140 ± 4	46 ± 2	212 ± 2
	Control	8	192 ± 3	130 ± 2	151 ± 2	61 ± 1	244 ± 1
	Control	9	159 ± 5	112 ± 2	127 ± 3	47 ± 3	187 ± 3
	>1-hr post	3	145 ± 2	108 ± 2	120 ± 2	37 ± 1	140 ± 4
	>1-hr post	4	116 ± 4	77 ± 2	90 ± 3	40 ± 2	197 ± 1
	>1-hr post	5	186 ± 5	98 ± 2	127 ± 3	88 ± 3	241 ± 1
	>1-hr post	6	142 ± 2	106 ± 2	118 ± 2	36 ± 1	173 ± 1
	>1-hr post	7	122 ± 4	74 ± 3	90 ± 4	48 ± 1	170 ± 2
	>1-hr post	8	155 ± 5	103 ± 4	120 ± 4	52 ± 2	237 ± 1
	>1-hr post	9	154 ± 3	99 ± 4	117 ± 3	56 ± 1	131 ± 4
	10-min post	5	157 ± 3	106 ± 2	123 ± 2	51 ± 2	224 ± 1
	10-min post	6	147 ± 2	112 ± 2	124 ± 2	35 ± 1	191 ± 1
	10-min post	7	124 ± 2	83 ± 1	96 ± 1	41 ± 1	155 ± 1
	10-min post	8	130 ± 4	92 ± 4	105 ± 4	37 ± 1	214 ± 1
	10-min post	9	119 ± 2	64 ± 2	83 ± 2	55 ± 1	116 ± 3
**Average**	Control		175 ± 23	124 ± 14	141 ± 17	51 ± 11	214 ± 20
	>1-hr post		146 ± 23 *	95 ± 14 **	112 ± 15 **	51 ± 18	184 ± 43 *
	10-min post		135 ± 16 **	92 ± 19 **	106 ± 18 **	44 ± 9	180 ± 44 *

Tukey’s HSD test used to compare timepoints.

* = p < 0.05,

** = p < 0.01,

*** p < 0.001.

n = 7.

Cardiovascular parameters were also recorded during this baseline eupneic breathing period before the cough trial. Cerebellectomy had a significant effect on systolic pressure [F(2,10) = 12.658; *p* = 0.002], diastolic pressure [F(2,10) = 17.724; *p* = 0.001], mean arterial pressure [F(2,10) = 17.57; *p* = 0.001], and heart rate [F(2,10) = 10.158; *p* = 0.004]. A post hoc analysis showed significant decreases at 10 minutes post-cerebellectomy compared to control: a 23% reduction in systolic pressure (*p* = 0.001), a 26% reduction in diastolic pressure (*p* = 0.001), a 25% decrease in mean arterial pressure (*p* = 0.001), and an 16% decrease in heart rate (*p* = 0.02). These effects persisted at the >1 hour timepoint, with a 17% decrease in systolic pressure (*p* = 0.02), a 23% decrease in diastolic pressure (*p* = 0.001), a 21% decrease in mean arterial pressure (*p* = 0.002), and a 14% decrease in heart rate (*p* = 0.04) when compared to control. Pulse pressure was unaffected by cerebellectomy at either timepoint [F(2,10) = 1.144; *p* = 0.4].

Table 3 is a correlation matrix showing all Pearson Product Moment Correlations for EMG amplitude, phase timing, and pressure measures during cough. These values are consistent with previous publications [25,27] with the cough total cycle time being primarily correlated with CT_E2_. There were also strong correlations between EMGs magnitudes for the diaphragm with the parasternal (r = 0.82), internal oblique (r = 0.61) and the PCA (r = 0.78) muscles. Additionally, expiratory esophageal pressures were strongly correlated with both the internal oblique (r = 0.65) and the PCA (r = 0.66) muscle EMG amplitudes.

**Table 3:** Pearson product moment correlations for all dependent measures across all conditions.

		**EMG Magnitude**					**Phase Timing**					**Esophageal Pressure**	
		**Diaphragm**	**Parasternal**	**Internal Oblique**	**PCA**	**Thyroarytenoid**	**CT**_**I**_	**CT**_**E**_	**CT**_**E1**_	**CT**_**E2**_	**CT**_**TOT**_	**I pressure**	**E pressure**
**EMG Magnitude**	**Diaphragm**		***0*.*82***	**0.61**	**0.78**	*0*.*54*	0.08	0.22	0.09	0.20	0.20	*-0*.*56*	*0*.*57*
	**Parasternal**			*0*.*57*	*0*.*47*	*0*.*52*	-0.04	0.07	0.10	0.04	0.03	*-0*.*58*	0.45
	**Internal Oblique**				*0*.*53*	*0*.*45*	-0.02	-0.04	-0.17	0.01	-0.05	-0.27	*0*.*65*
	**PCA**					0.26	-0.17	0.05	0.08	0.03	-0.05	*-0*.*56*	*0*.*66*
	**Thyroarytenoid**						<-0.01	0.10	-0.15	0.16	0.07	-0.07	0.23
**Phase Timing**	**CT**_**I**_ **(ms)**							0.27	0.05	0.26	*0*.*68*	0.19	-0.12
	**CT**_**E**_ **(ms)**		0.0–0.2	Neglible					0.20	***0*.*96***	***0*.*89***	-0.13	0.20
	**CT**_**E1**_ **(ms)**		0.2–0.4	Weak						-0.10	0.17	-0.44	-0.04
	**CT**_**E2**_ **(ms)**		0.4–0.6	*Moderate*							***0*.*85***	<0.01	0.21
	**CT**_**TOT**_ **(ms)**		0.6–0.8	**Strong**								<-0.01	0.09
**Esophageal Pressure**	**I pressure**		0.8–1.0	***Very Strong***									*-0*.*49*
	**E pressure**												

n = 9.

## Discussion

While the cerebellum is important for processing motor feedback, its specific role in cough has not been entirely elucidated. Our results demonstrate a short-term (10 minutes after cerebellectomy) enhancement in laryngeal adduction and inspiratory related amplitudes generated during cough, with no effect on phase durations or cough frequency. However, by the >1 hour timepoint, there was stark animal-by-animal variation in the effect of cerebellectomy on cough (Fig 2). This unpredictability suggests that cerebellar modulation of cough and respiratory related behaviors cannot be assumed to comprise merely simple motor feedback.

### Cerebellectomy produced time-dependent changes on cough magnitude

Our data differ from those reported by Xu et al. [20]; we observed no significant change in the number, frequency, or phase timing of cough, nor did we notice any consistent depression in motor output. Acutely, there was a significant increase in cough motor drive, but at ~1 hour post-cerebellectomy there were large variations between animals. The absence of a paralytic agent in our animals is one possible explanation for the differences reported between here and in Xu et al [20]. In our animals, the cerebellum likely received the dynamic muscle-related feedback from large chest wall excursions during cough and may have exerted an inhibitory effect on some aspect of the cough pattern generator or medullary bulbospinal premotor neurons. Additionally, our preparation is cerebrally intact, preserving suprapontine pathways that connect the brainstem with midbrain and forebrain structures. We also cannot rule out the possibility that the differences between our results and those of Xu et al. [20] can be explained at least in part by the lack of suprapontine pathways. Suprapontine structures are involved in sensation of tussigenic stimuli and cough production [33,34]. Studies of higher order brain structures and cough typically focus on evoked or volitional cough, but some report evidence that suprapontine brain regions and altered cognitive states modulate reflex cough [35–37]. Suprapontine pathways can also affect cerebellar function, [17,38–40] possibly obfuscating the influence of the cerebellum on cough. Finally, differences in anesthesia may contribute to disparate study results. The role of anesthesia in modifying cough is not fully understood. However, intense coughing occurs in our preparation and expiratory esophageal pressures can exceed 100 mmHg [41], which is within the range of that produced in awake humans [42]. This observation is consistent with a limited direct effect of pentobarbital anesthesia (maintained at a surgical level) on maximum cough motor drive in cat. Of note, neither our data nor those presented by Xu et al. [20] account for additional changes in cough due to craniotomy and the associated perturbations and potential dysregulation of intracranial pressure. The influence of cough on intracranial pressure has been well documented [43], however, to our knowledge no study has quantified the effects of craniotomy alone on cough.

Acutely, removing the cerebellum resulted in significantly stronger motor output during coughing when compared to control; this was followed by significant animal-by-animal variability at the >1 hour timepoint. The specific mechanism of this effect is unknown, but may it be mediated primarily through vagal or spinal feedback onto brainstem areas. Xu and Frazier [44] reported that cerebellectomy altered abdominal EMG and phase timing when respiratory drive was experimentally increased by expiratory loading and occlusion, and that these changes disappeared after vagal cooling but not dorsal column cooling. Vagally-mediated reflex responses to lung inflation are well-described as the result of pulmonary stretch receptors synapsing on pump cells in the respiratory control network [7,45,46]. It is plausible that bulbocerebellar brainstem connections, along with afferent information from the vagus, are the primary physiological mechanism for the influence of the cerebellum on cough, as opposed to spinocerebellar pathways.

Our results support the idea that the cerebellum, in animals with an anatomically intact nervous system, can regulate the gain of motor activation of upper airway and chest wall muscles during cough. Additionally, the effect on motor output became highly variable an hour after cerebellectomy, with animals displaying a heterogeneous response to cerebellectomy at this timepoint. Cough, as a behavior that is capable of large shifts in excitability, may have revealed a previously obscured feature of cerebellectomy. This variability may have been revealed by our experimental preparation because the animals are relatively more intact. In paralyzed, decerebrated preparations, mechanically-induced fictive cough-related inspiratory motor drive is approximately 65% greater than that during breathing [47]. However, in anesthetized animals with intact nervous systems, inspiratory motor drive during tracheobronchial cough can be increased by over 300% [48]. Therefore, inspiratory gain modulation during cough is more pronounced in anesthetized, unparalyzed preparations with an intact neuraxis. This motor regulatory mechanism is more similar to that observed in unanesthetized humans. This increased range for gain modulation in this preparation may underlie disparate reported effects of cerebellectomy. For example, cerebellectomy has been variously reported to increase [49], decrease [50], or have no effect [40,51–53] on respiration. These discrepancies are between studies rather than within one data set as presented here. Future investigations of the effects of cerebellectomy on the upper airway may need to report individual values in addition to group means to adequately represent the diversity of responses. The mechanism by which motor output shifted from a homogenous to heterogeneous response after cerebellectomy is unknown. It is possible that the effect on the tissue of the lesion itself caused an early increase in excitation, followed later by more heterogeneous responses upon reaching a new homeostasis. Future cerebellectomy studies may need to evaluate this perturbation over a longer time period and/or at a more granular level.

The present correlations are consistent with previous manuscripts [25,27], with limited relationships between the inspiratory and expiratory phases of cough and strong relationships between measures of expiratory duration (CT_E_ and CT_E2_) with total cough cycle time. In the present analysis we also expanded the variables of interest and were surprised that diaphragm amplitude had moderate relationships with internal oblique and thyroarytenoid muscle amplitudes. This may be due in part to the expanded data set, which includes increased and decreased states of excitability, and could warrant further study.

### Cerebellectomy slightly reduced respiratory rate due to a prolongation of the expiratory phase of breathing

Past investigations of cerebellectomy effects on eupneic breathing show mixed results. We noticed that cerebellectomy altered the eupneic ventilatory pattern, reducing respiratory rate primarily through the prolongation of the expiratory phase of breathing. This change was not accompanied by decreased end-tidal CO_2_. Williams et al. [50] similarly reported a prolonged T_TOT_ and T_E_ (in addition to a slightly prolonged T_I_) and a reduced respiratory frequency post-cerebellectomy in vagal intact animals. However, several other publications reported no change in eupneic breathing values in vagally intact animals [40,51–53]. At least one study did report increased respiratory frequency, but this sample included some vagotomized animals [49]. Furthermore, a change in respiratory frequency without a change in cough frequency is supported by our prior results, suggesting differential regulation of cough and breathing [48].

### Cerebellectomy decreased blood pressures and heart rate

We also observed modest decreases in blood pressures (systolic, diastolic, and mean arterial pressures) and heart rate post-cerebellectomy in the period immediately before a cough trial. Several of the aforementioned studies reported no change in blood pressure [40,52,53]. However, Williams et al. [50] reported that cerebellectomy or lesioning the fastigial nucleus reduced heart rate. Cerebellectomy can also reverse or reduce the increases in blood pressure produced by peripheral nerve stimulation in the rabbit and cat [54,55].

Several studies have demonstrated cerebellar connections with brainstem centers believed to be involved with autonomic regulation [56–58]. A fastigial pressor response [59–61] can be elicited by electrical stimulation of the fastigial nucleus via fastigiobulbar pathways in the brain stem. It is possible that this fastigial pressor response is active during bouts of repetitive coughing, and that by cerebellectomizing the animals, we removed part of a feedback loop tasked with maintaining a stable blood pressure during bouts of extreme thoracic pressure changes. Another potential source of this discrepancy between studies could be differences in anesthetic type or depth.

While we cannot completely discount the role of blood pressure on cough in our preparation, the effect of blood pressure on cough likely produces a separate cough phenotype [62]. In prior studies, reductions in blood pressure did enhance cough related EMGs, but it also produced changes in cough phase timing and an increase in cough number that we did not see post-cerebellectomy. Lutherer & Williams [63] were also able to elicit changes in breathing while pharmacologically blocking changes in cardiovascular output, providing further evidence for cerebellar influence on respiration independent of other autonomic changes.

### Laryngeal regulation during cough

Since the investigation of Poliacek and colleagues [26], techniques for laryngeal EMG placement for recording the adduction and abduction of the larynx have improved. The present study is the first to report motor drive to laryngeal motoneurons before and after cerebellectomy. Consistent with Poliacek et al. [26], we observed biphasic activity in the PCA (laryngeal abductor), and a depression in PCA activity concomitant with the thyroarytenoid (laryngeal adductor) burst (Fig 1). There is, however, some overlap in activity between the thyroarytenoid and PCA muscles as the cough shifts from the compressive to the expulsive phase. The coactivation of these muscles may act to stiffen the laryngeal apparatus during the extreme pressures generated during cough, and feedback from laryngeal muscle spindles may facilitate coordination between these muscles. Afferent projections from the larynx are represented in the cerebellum [16], and perhaps this feedback regulates motor output to maintain a set point for laryngeal stiffness during cough. Interestingly, we observed the opposite effect of cerebellectomy on thyroarytenoid activity during swallow reported in Reed and colleagues [51]. These data in combination with what is previously understood about breathing demonstrate a clear need for research on all pattern generators important for airway protection, as the effects on a single behavior are not translated to others.

## Conclusions

Shortly after cerebellectomy (10 minutes), there are significant increases in cough motor drive. After ~1 hour, this changed, as individual animals had highly variable responses during cough. However, the effect of the cerebellum on cough seems to be dependent on the presence of movement-related afferent feedback to the remainder of the respiratory/cough control network. Further research is necessary to determine which cerebellar structures and pathways are responsible for clinical disorders[21], and also to determine why cerebellar dysfunction can produce such variable effects [64,65]. These results suggest that, while the cerebellum and behavior-related sensory feedback regulate cough, it may be difficult to predict the nature of the modulation based on total cerebellectomy.

## Supporting information

S1 Checklist ARRIVE guidelines checklist. (PDF)

S1 Dataset Raw data. (CSV)
